# A glycosylation-related gene signature predicts prognosis, immune microenvironment infiltration, and drug sensitivity in glioma

**DOI:** 10.3389/fphar.2023.1259051

**Published:** 2024-01-16

**Authors:** Yanbo Yang, Haiying Teng, Yulian Zhang, Fei Wang, Liyan Tang, Chuanpeng Zhang, Ziyi Hu, Yuxuan Chen, Yi Ge, Zhong Wang, Yanbing Yu

**Affiliations:** ^1^ China-Japan Friendship Hospital, Chinese Academy of Medical Sciences and Peking Union Medical College, Beijing, China; ^2^ Department of Neurosurgery and Brain and Nerve Research Laboratory, The First Affiliated Hospital of Soochow University, Suzhou, Jiangsu, China; ^3^ Suzhou Medical College of Soochow University, Suzhou, Jiangsu, China; ^4^ Department of Neurosurgery, China-Japan Friendship Hospital, Beijing, China; ^5^ Department of Neurosurgery, Peking University China-Japan Friendship School of Clinical Medicine, Beijing, China; ^6^ The Affiliated Changzhou Second People’s Hospital of Nanjing Medical University, Nanjing, Jiangsu, China

**Keywords:** glycosylation, glioma, tumor microenvironment, prognosis, immunity, drug sensitivity

## Abstract

Glioma represents the most common primary cancer of the central nervous system in adults. Glycosylation is a prevalent post-translational modification that occurs in eukaryotic cells, leading to a wide array of modifications on proteins. We obtained the clinical information, bulk RNA-seq data, and single-cell RNA sequencing (scRNA-seq) from The Cancer Genome Atlas (TCGA), Chinese Glioma Genome Atlas (CGGA), Gene Expression Omnibus (GEO), and Repository of Molecular Brain Neoplasia Data (Rembrandt) databases. RNA sequencing data for normal brain tissues were accessed from the Genotype-Tissue Expression (GTEx) database. Then, the glycosylation genes that were differentially expressed were identified and further subjected to variable selection using a least absolute shrinkage and selection operator (LASSO)-regularized Cox model. We further conducted enrichment analysis, qPCR, nomogram, and single-cell transcriptome to detect the glycosylation signature. Drug sensitivity analysis was also conducted. A five-gene glycosylation signature (*CHPF2*, *PYGL*, *GALNT13*, *EXT2*, and *COLGALT2*) classified patients into low- or high-risk groups. Survival analysis, qPCR, ROC curves, and stratified analysis revealed worse outcomes in the high-risk group. Furthermore, GSEA and immune infiltration analysis indicated that the glycosylation signature has the potential to predict the immune response in glioma. In addition, four drugs (crizotinib, lapatinib, nilotinib, and topotecan) showed different responses between the two risk groups. Glioma cells had been classified into seven lines based on single-cell expression profiles. The five-gene glycosylation signature can accurately predict the prognosis of glioma and may offer additional guidance for immunotherapy.

## Introduction

Histologically, there exist over 100 distinct primary brain and central nervous system tumors (Davis, 2018). Glioma is the most prevalent form of primary cancer in the central nervous system of adults, with an annual diagnosis rate of 2.96 cases per 100,000 individuals in the United States (Fogh et al., 2010), accounting for 15% of all brain tumors (Ostrom et al., 2018). However, despite surgery, radiotherapy, and chemotherapy, most gliomas inevitably recur (Barthel et al., 2019). Approximately 52%–62% of patients have a recurrence within 5 years. Patients with glioblastoma multiforme (GBM), the most malignant glioma (World Health Organization [WHO] grade IV), using the current standard of care, have an average lifespan of 14 months after diagnosis (Van Meir et al., 2010). There are still fatal prognoses and high rates of mortality and recurrence (Wang et al., 2017).

Glycosylation is one of the most abundant and diverse forms of post-translational modification of proteins in eukaryotic cells, where sugar molecules are attached to nascent proteins (Schjoldager et al., 2020). The formation of glycosidic bonds is a dynamic process regulated by various enzymes, such as glycosyltransferases and glycosidases. The nitrogen of asparagine (N-glycans) or the oxygen of serine or threonine (O-glycans) is usually responsible for attaching glycan chains to their polypeptide backbone in glycoproteins (Rodrigues et al., 2018). Glycosylation plays a crucial role in the molecular and cellular mechanisms of tumorigenesis. Tumor growth relies on cancer cells’ ability to bypass cellular division checkpoints, evade immune surveillance and death signals, and migrate to metastatic sites. Glycosylation plays a critical role in all of these processes (Reily et al., 2019). Although glycosylation has been implicated in various biological processes and diseases, the specific role of glycosylation in glioma remains a relatively unexplored area. Investigating the functional roles of the identified gene signature can provide insights into the underlying mechanisms through which glycosylation influences glioma development, invasion, and therapeutic response. In this study, we investigated the roles of core glycosylation genes in glioma and established a glycosylation gene model to predict glioma patient survival and tumor immune microenvironment, elucidating that the underlying correlation is of great clinical significance. To the best of our knowledge, this is the first paper to investigate the relationship between glioma and glycosylation, and we hope our research can fill the research gap and inspire others.

## Methods

### Data collection

We retrieved clinical and mRNA sequencing data from The Cancer Genome Atlas (TCGA, https://portal.gdc.cancer.gov/), Chinese Glioma Genome Atlas (CGGA, http://www.cgga.org.cn/), and Repository of Molecular Brain Neoplasia Data (Rembrandt, https://gtexportal.org/home/) databases. RNA sequencing data for normal brain tissues were obtained from the Genotype-Tissue Expression (GTEx) database. Raw data were collected from the corresponding databases, filtered, and normalized using the EDASeq package (Bullard et al., 2010) (version 3.15). Batch effects were corrected using the R package ComBat (Martin et al., 2015) (version 3.15).

### Univariate Cox regression analysis and differential expression analysis

We obtained 168 glycosyltransferase-related genes through public databases. Then, we performed univariate Cox regression with the genes of interest in TCGA and CGGA cohorts. Genes that showed an adjusted *p*-value of less than 0.05 were considered significantly correlated with survival. In addition, we conducted a differential expression analysis of gliomas and normal brain tissue using TCGA and GTEx datasets. The R package edgeR (Robinson et al., 2010) (version 3.15) was used to verify the expression fold change value (expressed as a logFC value) and statistical significance. Genes with an absolute logFC value greater than 1 and a false discovery rate less than 0.05 were regarded as differentially expressed.

### Construction and validation of the model

The overlapping genes were subjected to variable selection using a least absolute shrinkage and selection operator (LASSO)-regularized Cox model. We randomly divided TCGA dataset into a training set and a testing set with a ratio of 4:1. The C-index was used for model performance evaluation. A bidirectional step-wise variable selection was performed. We used the R packages glmnet (Simon et al., 2011) (version 4.1-4), StepReg (Variyath and Brobbey, 2020) (version 1.4.3), and survival (Bair and Tibshirani, 2004) (version 3.3-1) for fitting and establishing the survival model. The forest plot was performed using the R package “survminer.” The risk score for each patient was calculated as follows:

$$RiskScore=\sum_{i=1}^{n}\left(Coef*xi\right).$$

The median risk score served as the threshold for classifying patients into high- or low-risk groups. The model was then validated in TCGA testing set and externally validated through CGGA and Rembrandt sets. ROC curves and AUC values were generated using the R package timeROC (Blanche et al., 2013) (version 0.4).

### Construction and evaluation of a nomogram

A nomogram was constructed to clarify whether the risk score was an independent prognostic predictor of glioma. Univariate and multivariate Cox regression analyses were performed to identify the independent prognostic factors for TCGA and CGGA datasets. We investigated the probability of 1-, 3-, and 5-year OS rates of patients with glioma based on the glycosylation score combined with clinical information including tumor grade, patient age, MGMT methylation, and IDH mutations. Then, we constructed a nomogram using the regplot package (version 1.1). The calibration plot and ROC curve were also performed to assess the accuracy of the nomogram for OS prediction.

### Pathway enrichment analysis and GSEA

We conducted a differential analysis between high-risk and low-risk groups using edgeR. Genes that met the criteria of absolute logFC value > 1 and false discovery rate <0.05 were identified as differentially expressed. We then conducted Gene Ontology and KEGG pathway enrichment analyses for the top differentially expressed genes. In addition, GSEA was also conducted. The R package clusterProfiler (Wu et al., 2021) (version 3.15) was used for all the above-mentioned enrichment analyses. These enrichment analyses aimed to identify molecular mechanisms that indicate a worse prognosis between the two subgroups.

### Cell line culture, qPCR, and IHC

We used quantitative polymerase chain reaction (qPCR) to detect glycosylation genes in cell lines. Glioma cell lines, namely, U87, T98G, A172, U251, HMC3 microglial cell line, and HA 1800 normal human astrocyte cell line, were obtained from ScienCell Research Laboratory, Cell Bank of the Chinese Academy of Sciences, and ATCC. The cells were cultured and maintained in DMEM supplemented with 10% fetal bovine serum. We extracted RNA using an mRNA extraction kit (Yeasen) following the instructions of the manufacturer. RNA was then reverse-transcribed. We used the qPCR SYBR Green Master Mix (Yeasen Biotechnology Co., Ltd., China) as a PCR indicator, and the qPCR process was performed using the QuantStudio Q5 Real-Time PCR System. The column plot was made using GraphPad Prism 8 software. After accessing the IHC images for each candidate glycosylation gene in glioma and normal tissue samples from The Human Protein Atlas database (HPA database, https://www.proteinatlas.org/), we assessed the staining intensity of each tissue according to the HPA database standards.

### Mutation burden and waterfall plot

We acquired the single nucleotide variation data with aliquot ensemble somatic variant merging and masking workflow from TCGA database through the TCGAbiolinks package (Colaprico et al., 2016). The mutation burden was calculated and then plotted using the ggbetweenstats package. The GenVisR package (Skidmore et al., 2016) was used to produce waterfall plots with the top 15 most commonly mutated genes in each group.

### Immune filtration

To study the immune infiltration level of gliomas, we applied the ESTIMATE algorithm (Yoshihara et al., 2013) to predict the immune fraction and stromal fraction for each sample. In addition, the fraction of each major immune cell was predicted and calculated through the xCell (Aran et al., 2017) algorithm. We created a heatmap to assess the differences between the high-risk and low-risk groups in the abundance of 32 immune cells and the stromal score, immune score, ESTIMATE score, and tumor purity.

### Drug sensitivity analysis

Cancer Cell Line Encyclopedia (CCLE) and Drug Sensitivity in Cancer v2 (GDSC2) were downloaded through the “PharmacoGx” package (Smirnov et al., 2016). This package enables efficient implementation of curated annotations of compounds, cell lines, and molecular features, facilitating integration and comparisons between different pharmacogenomic datasets. *In vivo* drug responses in cancer patients were predicted using the “oncoPredict” package (Maeser et al., 2021). The calcPhenotype function is capable of predicting the half-maximal inhibitory concentration (IC_50_) of drugs for glioma patients by fitting a ridge model, in which the testing sets are RNA-seq profiles of glioma patients in TCGA or CGGA and the training sets are the gene expression profiles of tissues and IC_50_ of the cancer cell lines to drugs from GDSC2 and CCLE. Drugs with the consistent direction of response (resistant or sensitive) in different combinations of training and testing sets were finally selected. The IC_50_ values from experiments of selected drugs between different tissues were then visualized using PharmacoDB 2.0 (https://pharmacodb.ca/) (Feizi et al., 2022).

### scRNA-seq data processing and cell–cell communication of glioma samples

Single-cell transcriptomes of glioma samples were downloaded from the GEO database (https://www.ncbi.nlm.nih.gov/geo/) (GSE167960). One WHO grade IV glioma and five WHO grade III gliomas were used for the analysis of cell populations and the expression of the markers of glial lineage. A total of 28,298 cells were analyzed using the R package “Seurat.” The FindIntegrationAnchors and IntegrateData functions were applied to remove the batch effect among different samples. Cells were clustered using FindNeighbors and FindClusters functions with a resolution of 0.5. The uniform manifold approximation and projection (UMAP) algorithm was used to reduce dimensionality. Cell types were identified through the use of marker genes from the CellMarker database (http://xteam.xbio.top/CellMarker). We used the “CellChat” packages to create CellChat objects. Then, the major ligand–receptor interactions in humans were evaluated using the “CellChatDB.human” database to perform the analysis of intercellular communication networks from annotated scRNA-seq data for seven cell clusters in all glioma samples. We then conducted intercellular communication networks on tumor-associated pathways.

## Results

### Identification and construction of a glycosylation gene prognostic model

**FIGURE 1:**
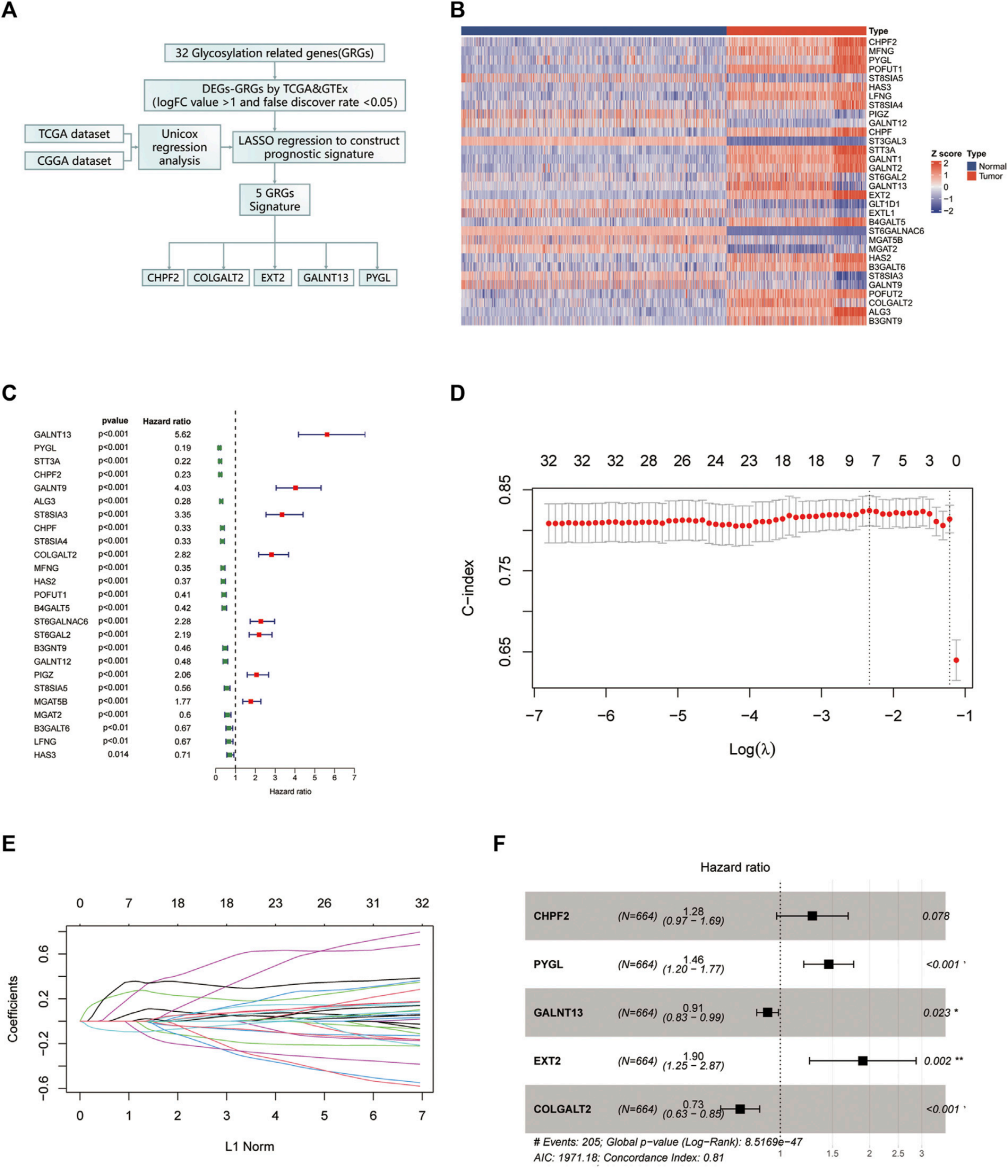
Identification of the glycosylation gene signature using LASSO-Cox regression. **(A)** Flow chart of our study. **(B)** The protein–protein interaction network was performed on the Metascape website. **(C)** Gene Ontology enrichment analysis was performed on the Metascape website. **(D)** Heatmap of all glycosylation genes between the normal brain and glioma tissues. **(E)** Forest plot of univariate Cox regression of differentially expressed glycosylation genes. **(F)** LASSO was used to conduct the log (lambda) sequence plot of glycosylation genes. **(G)** LASSO coefficient profiles of glycosylation genes in TCGA training dataset. **(H)** Forest plot of five glycosylation signature genes. LASSO, least absolute shrinkage and selection operator; TCGA, The Cancer Genome Atlas. **p < 0.01, ***p < 0.001.

The flow chart of our study is shown in Figure 1A. First, we chose genes that are differently expressed in normal brain tissue and glioma using normal brain tissue expression data from the GTEx dataset and glioma expression data from TCGA dataset. Genes with an absolute logFC value greater than 1 and a false discovery rate below 0.05 were identified as differentially expressed. The gene expression heatmap is shown in Figure 1B. Univariate Cox regression analysis was conducted to investigate prognostic-related gene analyses using TCGA dataset. We chose 32 glycosylation genes shared by the three datasets. The forest plot of the selected genes is shown in Figure 1C. Then, we performed a LASSO regression with fit measured by the C-index. The model fit across different lambda values is shown in Figure 1D. The coefficient plot is shown in Figure 1E. The selected five-gene model with their corresponding HR value is plotted in Figure 1F. The Venn plot of overlapping genes between TCGA, CGGA, and GTEx datasets is shown in Supplementary Figure S1.

#### The relationship between the expression status of the glycosylation gene signature and the tumor grade and prognosis

**FIGURE 2:**
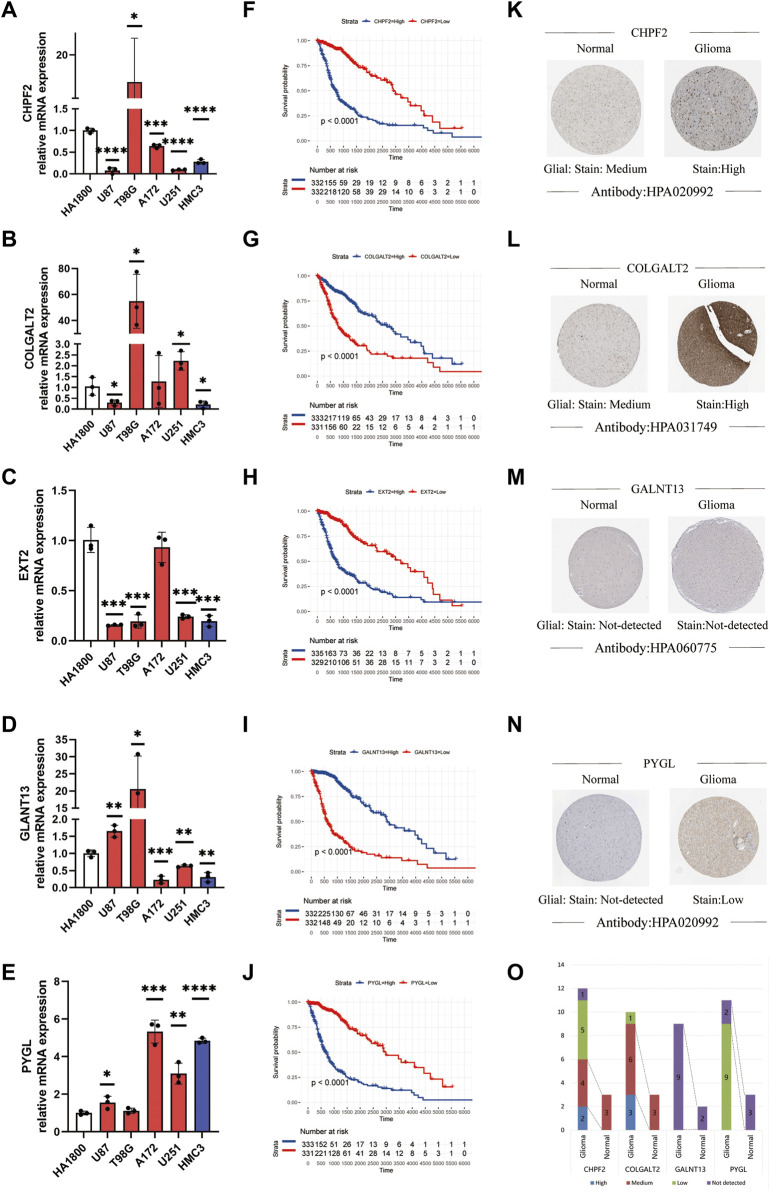
Relationship between the tumor grade and prognosis and the expression status of the glycosylation gene signature. **(A–E)** Expression level of mRNA of the glycosylation gene signature in different cell lines using qPCR. (*p < 0.05, **p < 0.01, ***p < 0.001, ****p < 0.0001, without label: no significant difference between the cell line and HA 1800). **(F–J)** Kaplan**–**Meier survival curves of different glycosylation gene signatures in glioma patients. **(K–N)** Protein expression images of different glycosylation gene signatures in the HPA database. **(O)** Histogram of glycosylation gene signature-related protein expression levels in the HPA database. qPCR, quantitative polymerase chain reaction; HPA, Human Protein Atlas.

We assessed the correlation between the expression status of the five genes and both tumor grade and prognosis. As to prognosis, higher expression of *CHPF2*, *PYGL*, and *EXT2* was associated with a worse prognosis, while that of *COLGALT2* and *GALNT13* was associated with a better prognosis. Furthermore, the mRNA expression of the five genes was measured in different cell lines using qPCR assays. The RNA expression levels were determined in glioma cell lines to verify our result. Five cell lines were used to replicate experiments and improve reliability. The results are shown in Figures 2A–E. The Kaplan–Meier survival curves of each gene in TCGA dataset are shown in Figures 2F–J. The Kaplan–Meier survival curves of the five genes are shown in Supplementary Figure S2. The Kaplan–Meier survival curves of each gene in the CGGA dataset (Supplementary Figures S3A–F) and REMx dataset (Supplementary Figures S4A–F) were made to further evaluate the relationship between the selected genes and prognosis. Moreover, we investigated the protein expression levels in glioma patients obtained from the Human Protein Atlas database and found greater staining intensity in glioma compared to normal brain tissues (Figures 2K–N). The corresponding statistical histogram is shown in Figure 2O.

### Construction and validation of the prognostic glycosylation genes

**FIGURE 3:**
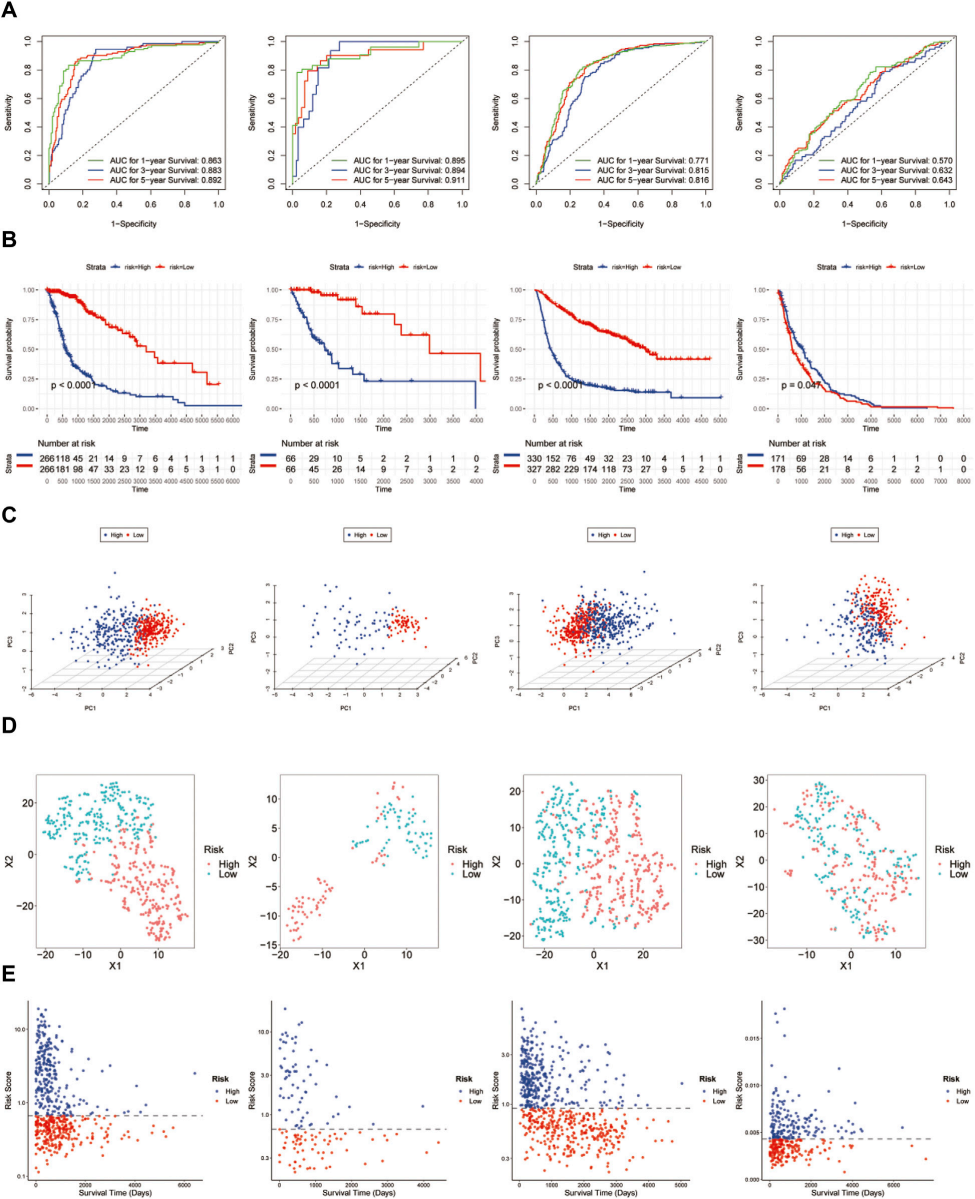
Prognostic risk model analysis of the glycosylation gene signature in TCGA-train, TCGA-test, CGGA, and Rembrandt datasets. **(A)** ROC curves demonstrate the predictive efficiency of the glycosylation gene signature on the 1-, 3-, and 5-year survival rates in TCGA-train, TCGA-test, CGGA, and Rembrandt datasets. **(B)** Survival curve of the glycosylation gene signature in these datasets. **(C)** PCA of the glycosylation gene signature in these datasets. **(D)** t-SNE analysis of the glycosylation gene signature in these datasets. **(E)** Distributions of survival time, survival status, and glycosylation risk score in these datasets. TCGA, The Cancer Genome Atlas; CGGA, Chinese Glioma Genome Atlas; PCA, principal component analysis; t-SNE, t-distributed stochastic neighbor embedding.

Based on the constructed signature, which is the summing up of each selected glycosylation gene expression level and corresponding coefficients, all the glycosylation scores of patients were calculated. Afterward, the patients with glioma were divided into high- and low-risk categories using the median glycosylation score as the threshold value (Figure 3A). The heatmap of the five selected genes between the high- and low-risk groups at the CGGA (Supplementary Figure S5) and TCGA (Supplementary Figure S6) datasets was used to screen the differential gene expression. The Kaplan–Meier survival curves indicate that the overall survival (OS) of the low-risk group patients in the CGGA cohort was significantly longer than that of the high-risk group patients. (*p* < 0.001). Subsequently, the same analyses were performed on TCGA-test cohort as well as in Rembrandt databases. The study revealed a noteworthy difference (*p* < 0.001) between the low-risk and high-risk groups (Figure 3B). PCA (Figure 3C), t-SNE analysis (Figure 3D), and the distribution plot of survival time, survival status, and glycosylation risk score (Figure 3E) also confirmed that the prognostic glycosylation genes could stably and accurately predict the prognosis of glioma patients.

### Evaluation of the correlation between glycosylation gene risk score and clinicopathologic characteristics

**FIGURE 4:**
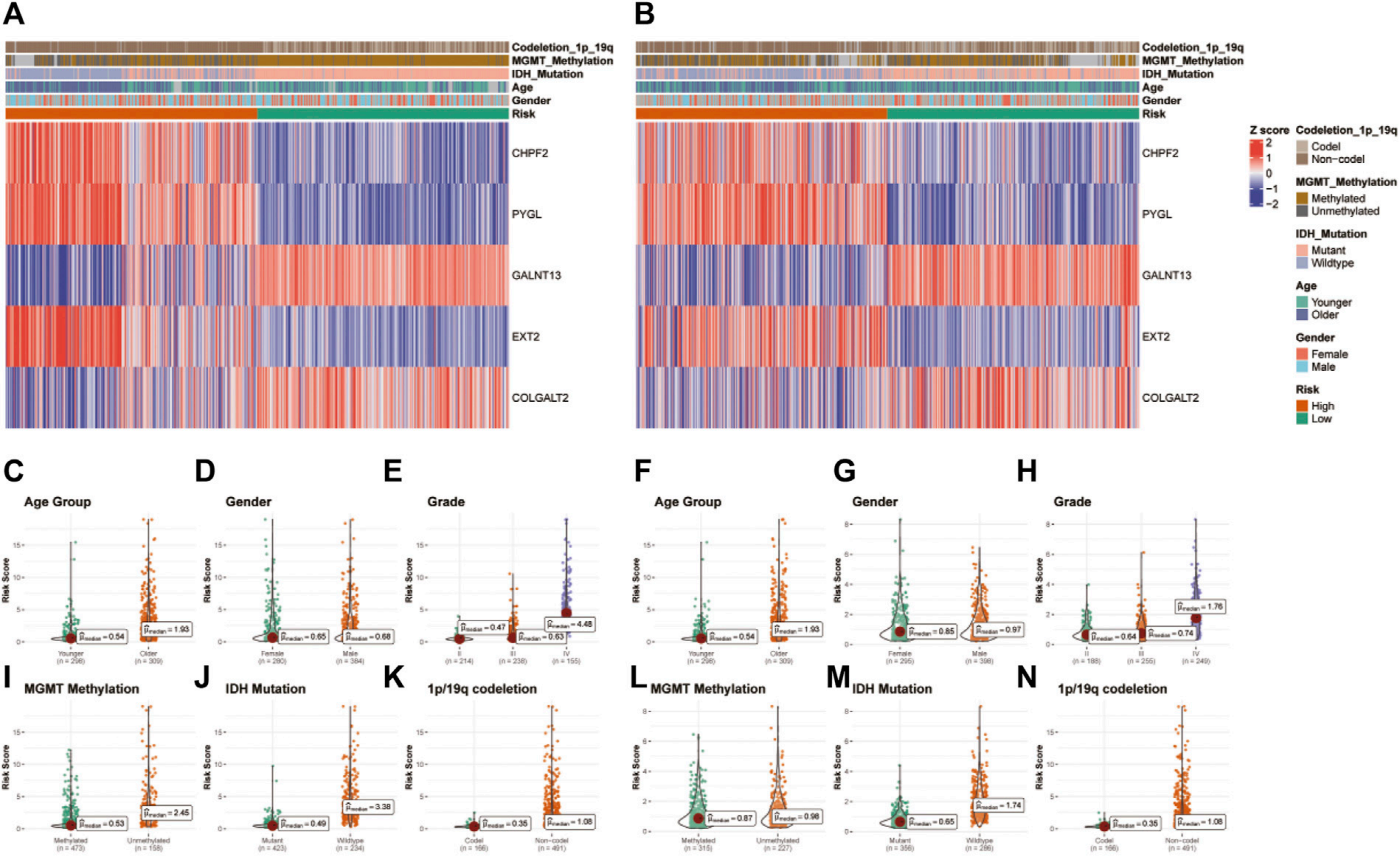
Correlation between the glycosylation gene risk score and clinicopathologic characteristics. **(A)** Heatmap of correlation between glycosylation gene risk groups with age, gender, tumor grade, MGMT methylation, IDH mutation, and 1p19q co-deletion and glycosylation signature gene expression in TCGA dataset. **(B)** Heatmap of correlation between glycosylation gene risk groups with age, gender, tumor grade, MGMT methylation, IDH mutation, and 1p19q co-deletion and glycosylation signature gene expression in the CGGA dataset. **(C–K)** Box and scatter plots of relationship between risk score and age **(C)**, gender **(D)**, tumor grade **(E)**, MGMT methylation **(I)**, IDH mutation **(J)**, and 1p19q co-deletion **(K)** in TCGA dataset and age **(F)**, gender **(G)**, tumor grade **(H)**, MGMT methylation **(L)**, IDH status **(M)**, and 1p19q co-deletion **(N)** in the CGGA dataset. TCGA, The Cancer Genome Atlas; CGGA, Chinese Glioma Genome Atlas; MGMT, O^6^-methylguanine-DNA methyltransferase; IDH, isocitrate dehydrogenase.

Heatmaps depict the correlation between risk groups of glycosylation genes and various clinical characteristics in TCGA (Figure 4A) and CGGA (Figure 4B) datasets. Violin plots indicate that the glycosylation risk score is highly related to these important clinicopathologic characteristics (Figures 4C–N), including age, gender, tumor grade, MGMT methylation, IDH mutation, and 1p19q co-deletion in TCGA and CGGA datasets.

### Establishment and evaluation of a nomogram based on independent prognostic factors for OS

**FIGURE 5:**
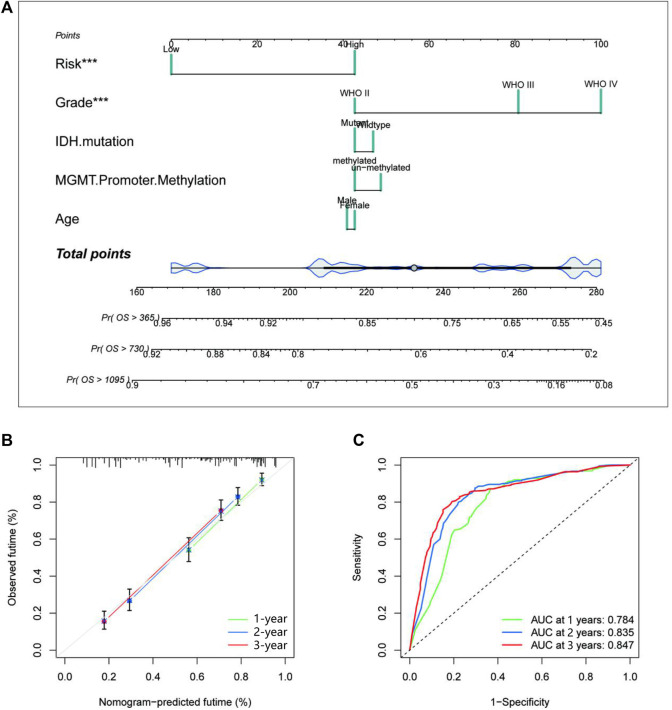
Establishment and evaluation of a nomogram in TCGA cohort. **(A)** The nomogram with glycosylation gene signature risk group for the prediction of OS of glioma patients was constructed based on TCGA dataset. **(B)** The time-dependent ROC analyses show the concordance between the predicted and observed 1-, 2-, and 3-year OS rates of the glycosylation gene nomogram in TCGA. **(C)** The calibration plots for predicting 1-, 2-, and 3-year survival using TCGA. TCGA, The Cancer Genome Atlas; OS, overall survival.

The study performed univariate and multivariate Cox regression analyses to identify glycosylation signatures as independent prognostic OS-related factors of glioma patients in TCGA database (Figures 5A–C). Then, a nomogram of TCGA cohort (Figure 5A) based on clinical characteristics, including WHO grade, IDH mutation, MGMT promoter methylation, age, and glycosylation score, was established. The calibration plots presented a concordance between the predicted probabilities from the nomogram and the observed 1-, 2-, and 3-year OS rates in TCGA cohort (Figures 5B, C). It is crucial that the nomogram has the ability to serve as a quantitative tool for forecasting survival outcomes in glioma patients. Our nomogram attained a greater net benefit than the single independent clinical feature. Meanwhile, the efficiency of the prognostic nomogram was clarified from multiple aspects.

### Functional enrichment analyses

**FIGURE 6:**
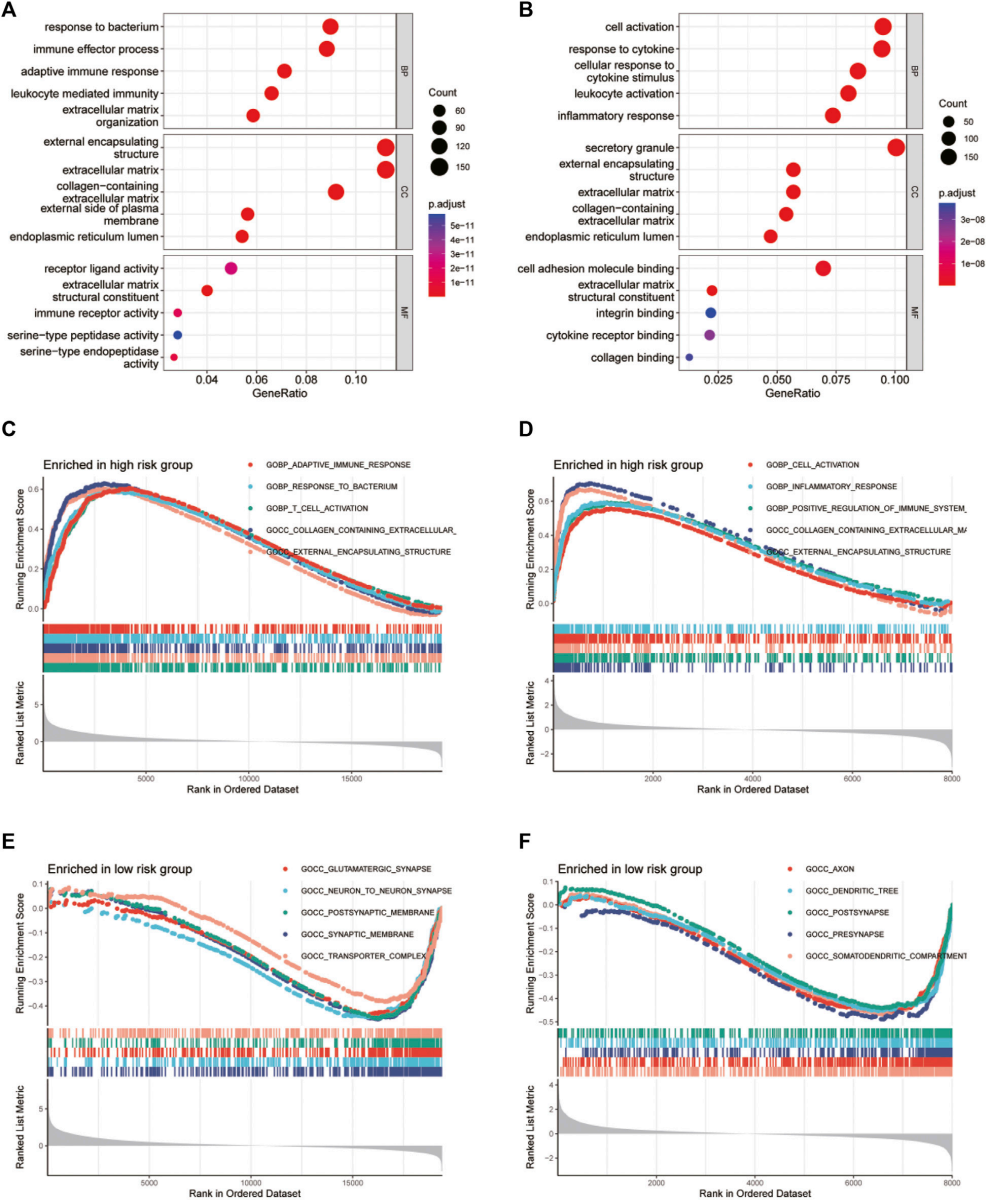
GO functional enrichment analyses between different glycosylation gene risk groups. **(A,B)** Bubble graph of GO enrichment analysis between high- and low-glycosylation gene risk group using TCGA **(A)** and CGGA **(B)**. **(C–F)** GSEA GO analyses of different glycosylation gene risk groups in TCGA or CGGA datasets. **(C)** high-risk, TCGA; **(D)** high-risk, CGGA; **(E)** low-risk, TCGA; **(F)** low-risk, CGGA. GO, Gene Ontology. TCGA, The Cancer Genome Atlas; CGGA, Chinese Glioma Genome Atlas; GSEA, gene set enrichment analysis.

To further clarify the functional mechanism of glycosylation genes and prognosis of glioma patients, we performed GO and KEGG enrichment analyses to characterize the biological functions of DEGs between the low-risk and high-risk groups. GO analyses in TCGA database (Figure 6A) revealed significant enrichment of biological processes, including response to the bacterium and immune effector process, and that in the CGGA database presented gene enrichment in immune regulation, including cell activation and response to cytokine (Figure 6B). At the same time, the KEGG pathway analysis revealed the enrichment of immune-related pathways in both databases (Supplementary Figures S7, S8). To confirm these results, we conducted GSEA in both the CGGA and TCGA datasets. We identified significant enhancement of multiple immune processes in the high-risk group in both cohorts, for example, adaptive immune response and response to the bacterium in TCGA cohort (Figure 6C) and cell activation, response to the bacterium, and positive regulation of the immune system in the CGGA cohort (Figure 6D). In addition, multiple neuron-related signatures were enriched in the low-risk group (Figures 6E, F).

### Immune filtration and tumor microenvironment

**FIGURE 7:**
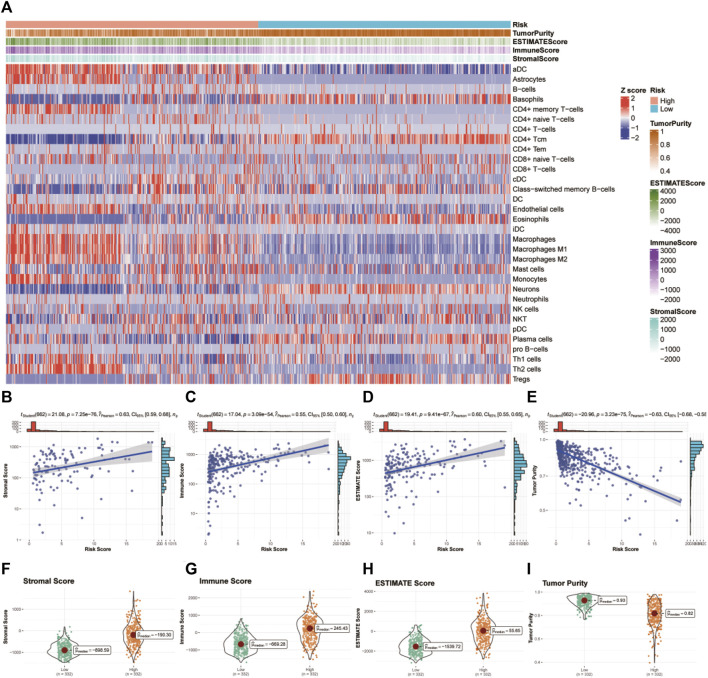
Tumor microenvironment and immunocorrelation analysis of glycosylation gene signature risk groups in glioma. **(A)** Heatmap of immune infiltration patterns between different risk groups in TCGA dataset. **(B–E)** Association of the risk score with the stromal score **(B)**, immune score **(C)**, ESTIMATE score **(D)**, and tumor purity **(E)**. **(F–I)** Correlation analysis between the risk score and stromal score **(F)**, immune score **(G)**, ESTIMATE score **(H)**, and tumor purity **(I)**.TCGA, The Cancer Genome Atlas.

We used the xCell algorithm to eliminate the cellular component of tumor samples from bulk sequencing data and analyzed the immune cell types between the high- and low-risk groups. In general, the high-risk group showed an enrichment of immune cells related to innate immunity, while the low-risk group showed an enrichment of adaptive immune cells (see Figure 7A). Then, we examined the correlation between glycosylation score and immune infiltration via stromal score, immune score, ESTIMATE score, and tumor purity. We found that the glycosylation score was significantly positively related to the stromal score, immune score, and ESTIMATE score and negatively related to tumor purity (Figures 7B–I), indicating that the glycosylation score was strongly correlated with local immune cell infiltration.

### Single nucleotide variation

**FIGURE 8:**
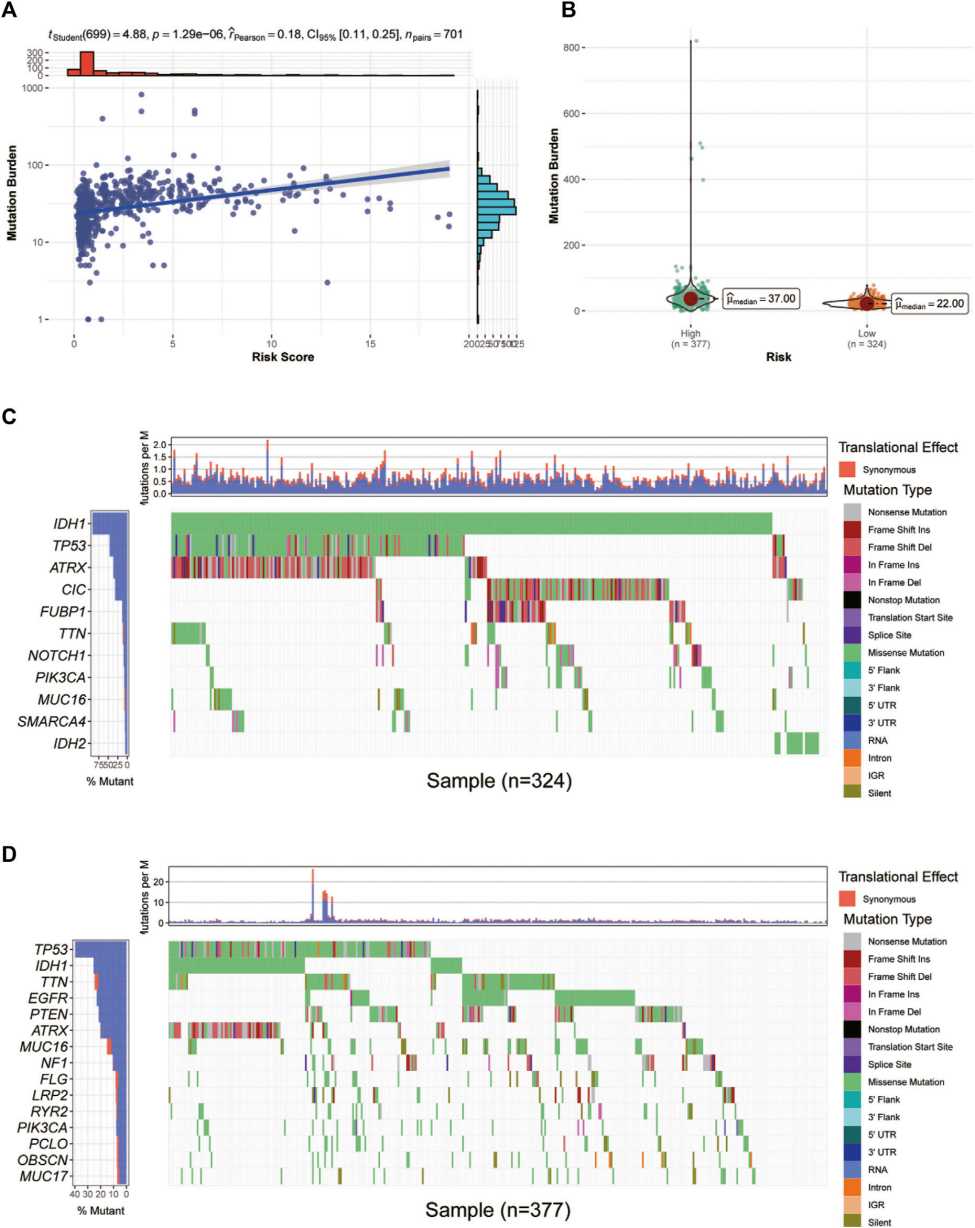
Correlation between glycosylation score and mutation burden in glioma patients in TCGA dataset. **(A,B)** Correlation analysis between glycosylation score and mutation burden. **(C,D)** The waterfall diagram demonstrates the top genes with the highest mutation frequency in the low- **(C)** and high-risk **(D)** groups. TCGA, The Cancer Genome Atlas.

First, we investigated the correlation between glycosylation score and mutation burden in glioma patients in TCGA cohort. As shown in Figure 8A, the glycosylation score was positively related to mutation burden. In addition, patients in the high-risk group have significantly increased mutation burden (Figure 8B). Then, we explored the specific mutation type and host genes in the high- and low-risk groups. In the low-risk group, the predominant mutation is the missense mutation of IDH1, which appeared in over 80% of all samples, followed by TP53 mutations, indicating a generally benign disease course (Figure 8C). However, in the high-risk group, the TP53 mutation is the most frequent mutation, accounting for only 40% of all mutations (Figure 8D).

### Identification of appropriate drugs for glioma patients in different risk groups

**FIGURE 9:**
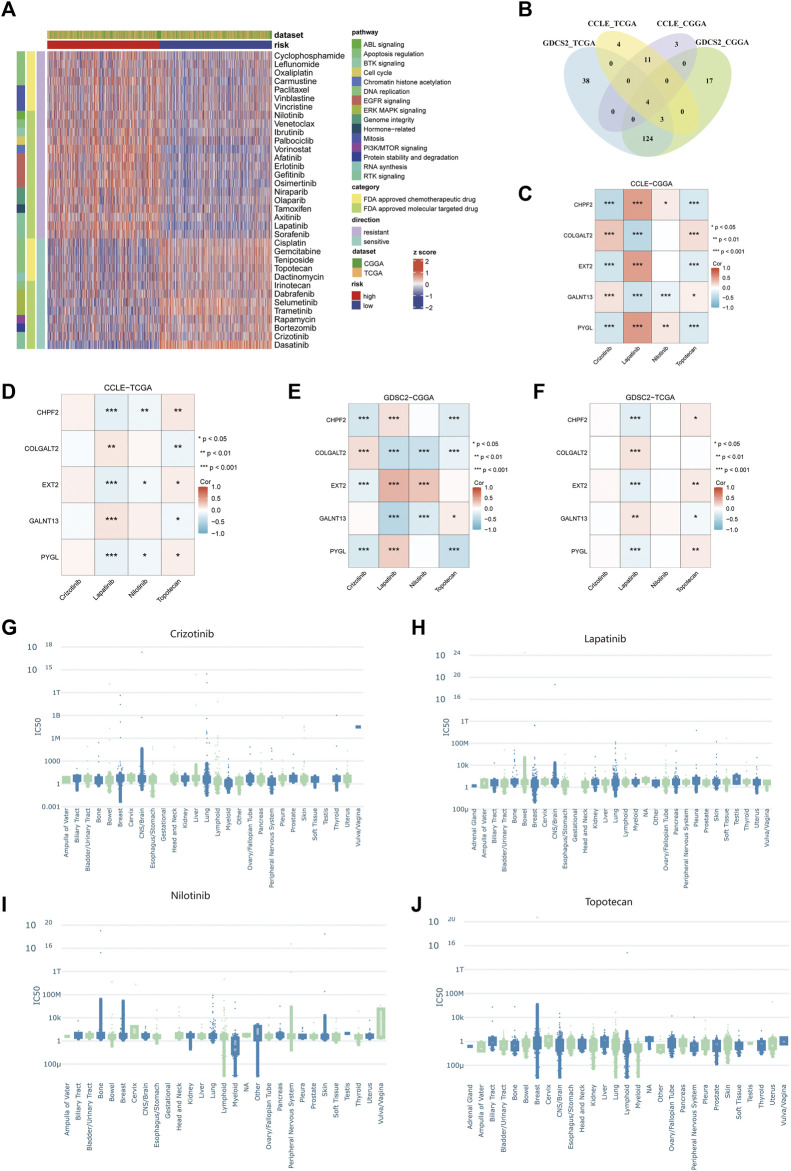
Prediction of drug sensitivity using the glycosylation signature in glioma patients. **(A)** The heatmap of predicted IC_50_ values of 35 FDA-approved drugs in GDSC2 between the high- and low-risk groups. **(B)** Venn diagram between different combinations of training and testing sets. **(C–F)** Correlation between the predicted IC_50_ values of four selected drugs and the expression of five glycosylation signatures among the combination of CCLE–CGGA **(C)**, CCLE–TCGA **(D)**, GDSC2–CGGA **(E)**, and GDSC2–TCGA **(F)**. **(G–J)**. The IC_50_ values from experiments of crizotinib **(G)**, lapatinib **(H)**, nilotinib **(I)**, and topotecan **(J)** among different cancer tissues in all available pharmacogenomic datasets in PharmacoDB.

To select appropriate drugs for patients in different risk groups, we used the oncoPredict algorithm to predict the IC_50_ value of glioma patients in TCGA and CGGA cohorts. In the GDSC2 pharmacogenomic dataset, 131 drugs were identified to show different responses in both TCGA and CGGA datasets, including 35 drugs that have been approved by the FDA (Figure 9A). Among them, 22 drugs were resistant in the high-risk group and 13 drugs were sensitive in the high-risk group. The pathways of these drug targets are shown in Figure 9A. Four drugs (crizotinib, lapatinib, nilotinib, and topotecan) have been identified after taking intersections in four databases (Figure 9B). To further explore the relationship between the predicted IC_50_ values of these four drugs and five glycosylation signatures, we performed Spearman’s correlation analysis in the permutation and combination of these four datasets. (Figures 9C–F). The IC_50_ values from experiments of these four drugs in different cancer tissues were visualized using PharmacoDB 2.0 (Figures 9G–J).

### Single-cell transcriptomes and cell–cell communication of glioma samples

**FIGURE 10:**
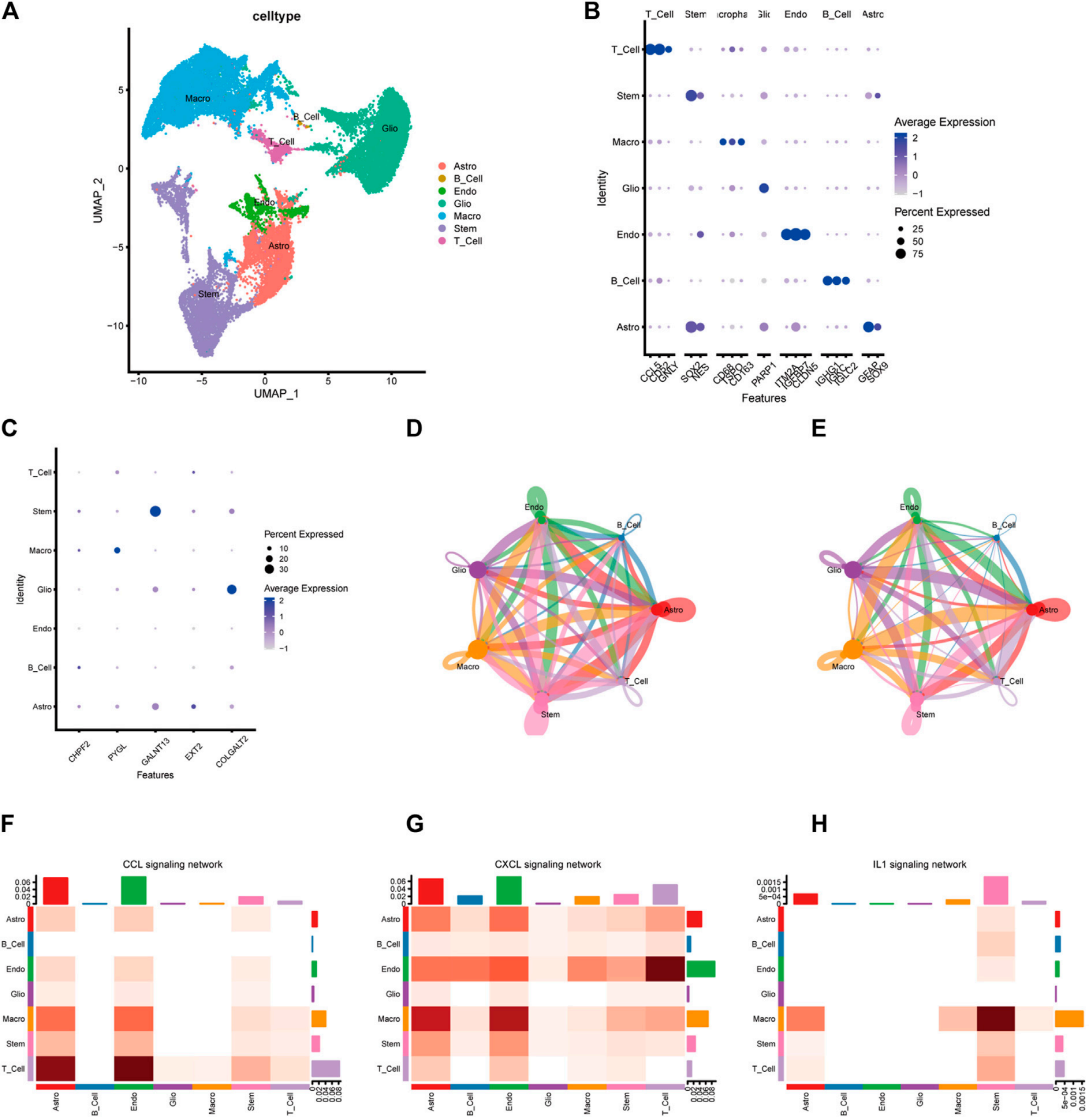
Single-cell transcriptome analysis of the five-gene glycosylation signature and cell–cell communication between different cell types. **(A)** Clustering and annotation of single-cell data. **(B)** Dot plot of the expression features of marker genes of the seven major clusters. **(C)** Identification of the five-gene glycosylation signature expression in seven cell types in glioma. **(D–E)** The circle interaction plots show the count **(D)** and weight **(E)** of inferred intercellular communication network analysis of glioma cells with the remaining several cell clusters. **(F–H)** The heatmaps show the communication probability on the CXCL signaling pathway **(F)**, CCL signaling pathway **(G)**, and IL1 signaling pathway **(H)** for different cell populations.

Glioma cells were classified into seven lines based on single-cell expression profiles: 1) astrocytes, 2) B cells, 3) endothelial cells, 4) glioma cells, 5) macrophages, 6) stem-like cells, and 7) T cells (Figure 10A). The expression features of marker genes of the seven major clusters are presented in Figure 10B. *GALNT13* was relatively highly expressed in stem-like cells and astrocytes. *COLGALT2* was highly found in glioma cells. *PYGL* was only highly found in macrophages. *CHPF2* and *EXT2* were relatively low in these seven cell lines (Figure 10C). We then explored the cell–cell interactions of glioma cells and different types of immune cells in glioma tissues (Figures 10D, E). The cell–cell communication networks showed that glioma cells do not have significant interactions with macrophages, T cells, and B cells. Heatmaps showed the communication probability for different cell populations on the CXCL signaling pathway (Figure 10F), CCL signaling pathway (Figure 10G), and IL1 signaling pathway (Figure 10H). Glioma may not have significant interactions with other cell types on these tumor-associated signaling pathways.

## Discussion

Gliomas are a heterogeneous assemblage of brain tumors that originate from genetically/epigenetically abnormal cells with glial stem/progenitor-like features (Nicholson and Fine, 2021). Although studies suggest that gliomas may be caused by mutations in genes such as TP53 (Pratt et al., 2022), their pathogenesis is not fully understood. Gliomas have traditionally been histopathologically classified as diffuse astrocytoma, oligodendrogliomas, and mixed astrocytoma/oligoastrocytoma based on the type of tumor cells (Lenting et al., 2017). In addition, gliomas are classified by malignancy grade (World Health Organization grades I–IV) based on marked mitotic activity, necrosis, and the presence or absence of erythematous microvascular hyperplasia (MVP) (Wesseling et al., 2015). Intratumoral heterogeneity is a well-known feature of gliomas and may result from selection pressures, such as nutritional limitation, clonal competition, and therapy (Sottoriva et al., 2013). The clinical manifestation of glioma depends on the anatomic sites, histopathology, and functional status. In newly diagnosed cases, the standard approach to treatment includes surgery, followed by concomitant radiotherapy with temozolomide and additional adjuvant temozolomide (Tan et al., 2020).

Glycosylation is a common, complex, and plastic post-translational modification of secreted and membrane-bound proteins. It is the outcome of a collaboration between nucleotide sugar transporters and biosynthesis pathways, along with glycosyltransferases and glycosidases present in the endoplasmic reticulum (ER) and the Golgi apparatus. Most of the final processing occurs in the cis-, medial-, and trans-Golgi compartments (Pinho and Reis, 2015). In these organelles, glycosyltransferases and glycosidases form carbohydrate structures through a series of stages that are controlled by enzyme activity, substrate availability, levels of gene transcription, and enzyme position within the organelles (Reily et al., 2019).

Glycans play a critical role in different stages of carcinogenesis, such as cancer cell dissociation and invasion, cell-to-cell adhesion, angiogenesis, immune modulation, and metastasis composition. Glycosylation is critical in cell communication and signaling (Haltiwanger and Lowe, 2004). Incomplete synthesis and neo-synthesis are two primary mechanisms that underlie carbohydrate-related structural changes in cancer, as identified by Kannagi et al. (2008), while the incomplete biosynthesis pathway is more common in the early stages of cancer. On the contrary, neo-synthesis typically occurs in the advanced stages of a tumor and is relevant to the induction of several genes involved in the glycosylation pathway (Kannagi et al., 2008). Glycosylation changes are commonly observed in cancer cells, including increased sialyl Lewis structures, irregular core fucosylation, an increase in N-glycan branching, or exposure of the mucin-type O-glycan, which may result in the production of unique tumor antigens that could be promising targets for immune therapy (Holmes et al., 1987). In some cases, malignant tissues triggered the recurrence of glycosylated antigens that were expressed in fetal life. Possibly the most extensively researched tumor-associated glycans consist of variations arising from a premature termination of protein O-glycosylation called the Tn antigen (the most basic O-glycan), its sialylated counterpart sialyl-Tn (sTn; Neu5Acα2-6GalNAcα-O-Ser/Thr), and the T or core 1 antigen which arises following the addition of a Gal residue to the Tn antigen (Galβ1-3GalNAc-Ser/Thr) (Julien et al., 2011). Both the sTn and sialylated T antigens are not present in normal healthy tissues but are overexpressed in many types of solid tumors (Marcos et al., 2011; Julien et al., 2012). The roles of glycans in the tumor have been highlighted by the fact that glycosylation variations are implicated in the growth and advancement of cancer, therefore serving as biomarkers and presenting a set of precise targets for therapy (Pinho and Reis, 2015). In our study, we found that the glycosylation score was positively correlated with immune infiltration, which may be interpreted by increased mutation burden for a high glycosylation score. This suggests that glycans play a role in immune infiltration in cancer cells.

Our study determined that glycosylation modification is crucial in tumor progression and has a strong correlation with glioma prognosis. The study selected prognostic-related genes based on univariate Cox regression across TCGA and CGGA and then filtered the overlapped genes through LASSO regression and step-wise Cox regression to build and validate a glycosylation-related gene signature through internal and external validation. The five genes are all involved in the glycosylation process. In the next paragraph, we will discuss the general function and the latest research progress of these genes through a systematic approach.

Chondroitin sulfate (CS) proteoglycan is a vital enzyme that is translated by the *CHPF2* gene. It exists in various tissues as a proteoglycan in which the disaccharide units of N-acetylgalactosamine and glucuronic acid residues are repeated, and the sulfate residues are distributed in different positions. This unique structure provides hyperosmolarity and water retention ability, and it may also play an important role in regulating cell adhesion to the extracellular matrix, thus acting as an important mediator for cell proliferation, cell migration, morphogenesis, and some cytokine signaling (Yada et al., 2003). Glycosaminoglycan plays a crucial role in morphogenesis and tissue development and contributes to tumor development and formation. The biosynthesis of CS is accomplished through a variety of enzymes, such as glycosyltransferases and sulfotransferases (Kalathas et al., 2011). Glycosaminoglycans are generally altered in tumors in qualitative and quantitative terms (Skandalis et al., 2007; Kalathas et al., 2009). In addition, the reduction in C-6 sulfation is more abrupt in cancer; the effect of C-4 sulfation is gradually diminished at the stage of advanced cancer. These changes may be caused by differences in core protein precursor biosynthesis, substrate pools, and expression level of the enzymes involved in chondroitin/dermatan sulfate biosynthesis (Kalathas et al., 2010). Compared to tumor tissues, adjacent macroscopic tissues have been observed to have lower levels of CS in many cancer types (Kalathas et al., 2011). A previous study found that the expression of *CHPF2* was positively correlated with the levels of macrophages and dendritic cells, which may influence drug sensitivity.

*PYGL* has been reported to have oncogenic effects in multiple tumors (Zhu et al., 2022). It has been reported to be a glycogenolysis-related gene whose expression is upregulated in various tumors (Zhao et al., 2021). The protein translated by *PYGL* is glycogen phosphorylase, liver form. Glycogen metabolism is considered a crucial pathway in cancer metabolism reprogramming (Yang et al., 2019). It was also believed that the degradation of glycogen, which is regulated by *PYGL*, could sustain proliferation and prevent premature senescence in cancer cells (Favaro et al., 2012). Zhu et al. (2022) revealed the relationship between the *PYGL* gene and the prognosis of glioma by analyzing the Chinese Glioma Genome Atlas database and using quantitative real-time PCR to verify the *PYGL* expression level in gliomas. Previous investigations have observed a comparatively sluggish rise in *PYGL* levels under hypoxic conditions. Further examination of *PYGL* expression in tumor cells has unveiled discernible patterns of induction in response to hypoxia (0.1% O_2_), which have been consistently observed at both the mRNA and protein levels. The result of the research indicated that *PYGL* could be regarded as a new biomarker and molecular target for assessing the prognosis and immunotherapy of glioma. Zhao et al. (2021) returned the same result. They found that the mRNA expression level of *PYGL* showed a positive correlation with the glioma grade. Overall survival and Cox regression analyses showed that high *PYGL* expression is an independent risk factor for a worse prognosis in glioma patients (*p* < 0.05).

*GALNT13* is one of the specialized glycosyltransferases termed polypeptide N-acetylgalactosaminyltransferases, which mediate the initial reaction in O-linked oligosaccharide biosynthesis. N-Acetylgalactosaminyltransferases catalyze the transfer of an N-acetyl-D-galactosamine (GalNAc) residue to a target protein at the serine or threonine residue, which will form the GalNAc-O-Ser/Thr structure, also known as Tn antigen (Raman et al., 2012). A prior study analyzed the expression levels of *GALNT13* mRNA in a variety of fetal and adult human tissues. The greatest expression level occurred in the fetal brain, trailed by the adult brain (Zhang et al., 2003). In the analysis of *GALNT13* mRNA expression levels among human cancer cell lines, Nogimori et al. (2016) discovered that the expression was higher only in neuroblastoma lines and lung tumors. Considering that *GALNT13* is basically expressed in the fetal brain, both *GALNT13* and its products of translation trimeric Tn may play an essential role in cell growth and proliferation and serve as a specific maker for malignant CNS tumors (Nogimori et al., 2016). Different exons in *GALNT13* mRNA exhibit distinct sequences in the lectin-like domain. Consequently, the contrasting outcomes observed in variant exon usages could potentially be attributed to variations in substrate recognition during O-glycan synthesis. Therefore, the utilization of tumor-specific and malignancy-associated variant exons may hold significance as targets for molecular therapy in cancer treatment, although further investigation is required to elucidate the precise mechanisms involved. Berois et al. (2006) found that *GALNT13* was the most highly expressed gene indicated by microarray gene expression analysis performed using a metastatic xenograft-derived cell model of human neuroblastoma, with a 12-fold upregulation in metastatic malignant neuroblasts compared to the primary cancer xenograft, suggesting that the *GALNT13* expression level could be potentially used to detect malignant neuroblasts at diagnosis or recurrence. However, it has not been investigated in clinical studies.

The exostosin (EXT) family of glycosyltransferases includes *EXT1* (located on chromosome 8q23-q24) and *EXT2* (located on chromosome 11p11-p12), which mediate the synthesis of heparan sulfate proteoglycans (HSPGs) (Busse-Wicher et al., 2014). HSPGs are ubiquitous component parts of the extracellular matrix and are involved in tissue homeostasis (Meneghetti et al., 2015). Numerous research studies have shown that heparin sulfate is essential for signal transduction in a variety of processes, such as cell survival, migration, division, differentiation, and cancer progression (Li and Kusche-Gullberg, 2016). Both genes (*EXT1* and *EXT2*) encoding exocrine glycosyltransferases have tumor-suppressive functions, although the details of mechanisms and predictions of the prognostic value of exostosin in cancer remain unclear. Ushakov et al. (2017) demonstrated a significant decrease in both *EXT1* and *EXT2* expression levels in gliomas. However, according to Wade et al. (2013), the expression levels of these genes are upregulated in glioblastoma tissues. The mutations in *EXT1* and *EXT2* may have a significant correlation with hereditary multiple exostoses (HME), a disorder dominantly associated with a genetic disorder characterized by the formation of multiple cartilaginous tumors. *EXT2* expression levels showed significant associations with tumor purity as well as infiltration levels of CD4^+^ T cells, macrophages, neutrophils, and dendritic cells in head and neck squamous cell carcinoma. The observed correlation between elevated *EXT2* expression and increased neutrophil infiltration in head and neck squamous cell carcinoma cases suggests that this phenomenon might be the most important factor associated with a poor prognosis. The germline heterozygous loss-of-function mutations in the tumor suppressor genes *EXT1* or *EXT2* shoulder the responsibility for over 70%–95% of HME cases. Al-Zayed et al. (2021) investigated genetic defects of *EXT2* in Saudi HME patients, and found that 77% of the patients had *EXT1* and *EXT2* mutations, while the *de novo EXT1* and *EXT2* mutations are popular.

The biological functions of human collagen beta (1-O) galactosyltransferase 2 (*COLGALT2*) in tumors have not been determined despite its significance in the collagen glycosylation process (Wang et al., 2020). Guo et al. (2021) found that compared with healthy ovary tissues, *COLGALT2* expression is significantly higher in both high‐grade and low‐grade serous ovarian cancer types, which are common subtypes of ovarian cancer. According to Wang et al. (2020), adipose-derived mesenchymal stem cell exosomes prompt the progression of osteosarcoma by increasing *COLGALT2* expression levels in osteosarcoma cells. The *COLGALT2* enzyme initiates post-translational glycosylation of collagen and is therefore a compelling target of osteosarcoma susceptibility (Kehayova et al., 2021). It was demonstrated that exosomes derived from adipose-derived mesenchymal stem cells promoted the invasion, migration, and proliferation of osteosarcoma cells. Furthermore, this effect was accompanied by an upregulation in the expression of *COLGALT2*.

Our study pooled multiple glioma datasets to build a universal model for all gliomas. However, the high degree of heterogeneity among different types of gliomas is one of the significant obstacles in the current treatment of glioma. Due to their shared origin in neuroepithelial-derived cells, LGG and GBM exhibit significant similarities in their malignant biological behavior. We tried to explore common biomarkers that can overcome this heterogeneity. Therefore, we included all types of gliomas in this study rather than focusing on specific types. In addition, combining the LGG and GBM datasets could increase the sample size in the predictive model, which may increase the overall accuracy and broaden the clinical usage of the model.

Functional enrichment analyses demonstrated differences in immune-related biological processes between the high- and low-risk groups. Additionally, the tumor immune microenvironment was investigated in both groups. It showed that the high-risk group had higher stromal, immune, and ESTIMATE scores and lower tumor purity. The high- and low-risk groups had different mutation burdens. Cancer-associated glycosylation may enhance the communication among tumor cells in a microenvironment through the glycan-binding receptor—lectin. Glycans are oligosaccharide structures discovered on proteins and lipids. Endogenous lectins are expressed on immune cells and additional cells in the stroma and facilitate cell–cell interactions and adhesion, thus contributing to homeostasis. During malignancy, alterations in the glycosylation of tumor cells may affect inflammatory responses, facilitate viral immune evasion, enhance cancer cell metastasis, or regulate apoptosis (Pinho and Reis, 2015). Crizotinib and topotecan were observed to be effective in the high-risk group, while lapatinib and nilotinib showed resistance in the same group. Notably, crizotinib, lapatinib, and nilotinib belong to the class of tyrosine kinase inhibitors (TKIs) that are commonly used in cancer therapy to target the epidermal growth factor receptor family (Roskoski, 2014). The application of TKIs to oncology was revolutionary. For instance, imatinib significantly increased the 5-year survival rate of Philadelphia chromosome-positive patients with chronic myelogenous leukemia (CML) from 11% to 90% (Bower et al., 2016). Topotecan is a water-soluble derivative of camptothecin that has antineoplastic activity in cell culture and xenograft systems. It has been approved as a second-line therapy in ovarian and small-cell lung cancer (SCLC). The drug disrupts the normal function of the nuclear enzyme topoisomerase I to inhibit the replication of rapidly dividing cells (Ormrod and Spencer, 1999).

Our study has several advantages. First, we constructed a glycosylation-related gene signature that includes only five genes yet shows high accuracy in the prognosis of glioma patients. Second, the gene signature was constructed based on a public database that contained a large number of patient samples. Third, no glycosylation-related gene signature has been found related to glioma; thus, this study fills the gap of glycosylation-related genes in the prognostic prediction of glioma. Our study built the predictive model based on high-throughput sequencing. Although it provided high-dimensional data with satisfying accuracy, it also limited the interpretation of our results. In clinical use, high-throughput sequencing of the tumor tissue is not widely used as a clinical routine. In addition, the use of normalization methods and the high tendency of batch effect may decrease the universal use of a model based on sequencing. In addition, the available samples for qPCR were not sufficient. Moreover, the underlying molecular mechanism has not been identified. Therefore, further investigations are required to study the interactions between glycosylation-related genes and glioma.

## Conclusion

This study developed a predictive model using a glycosylation signature. This model can accurately predict the survival outcomes of glioma patients and has been validated using external sources of data. Additionally, the glycosylation signature correlated with immune infiltration in the glioma microenvironment and may suggest varying levels of effectiveness for immunotherapy. These findings will offer valuable insights for future studies and clinical practice.

## Data Availability

The original contributions presented in the study are included in the article/Supplementary Material; further inquiries can be directed to the corresponding authors.
