# Supplementary material for: A glycosylation-related gene signature predicts prognosis, immune microenvironment infiltration, and drug sensitivity in glioma
Source: Front Pharmacol. 2024 Jan 16;14:1259051. doi: 10.3389/fphar.2023.1259051 (PMC10824914; doi:10.3389/fphar.2023.1259051)
Supplement: Supplementary file 2 [file DataSheet3.docx]

**Glycosylation-Related Gene Signature Predicts Prognosis and Indicates Immune Microenvironment Infiltration in Glioma**

**List of supplemental materials:**

**S1. The Venn diagram was used to screen overlapping genes between TCGA, CGGA and GTEx datasets.**

**S2. Kaplan–Meier survival analysis of glycosylation related gene signature in the TCGA cohort.**

**S3. Kaplan–Meier survival analysis of the each and overall glycosylation related gene signature in the CGGA cohort.**

**S4. Kaplan–Meier survival analysis of the each and overall glycosylation related gene signature in the REMx cohort.**

**S5. The heatmap was used to screen differential gene expression of 5 selected genes between the high- and low-risk groups at CGGA dataset.**

**S6. The heatmap was used to screen differential gene expression of 5 selected genes between the high- and low-risk groups at TCGA dataset.**

**S7. KEGG functional enrichment analysis of CGGA datasets.**

**S8. KEGG functional enrichment analysis of TCGA datasets.**

**S1. The Venn diagram was used to screen overlapping genes between TCGA, CGGA and GTEx datasets.**


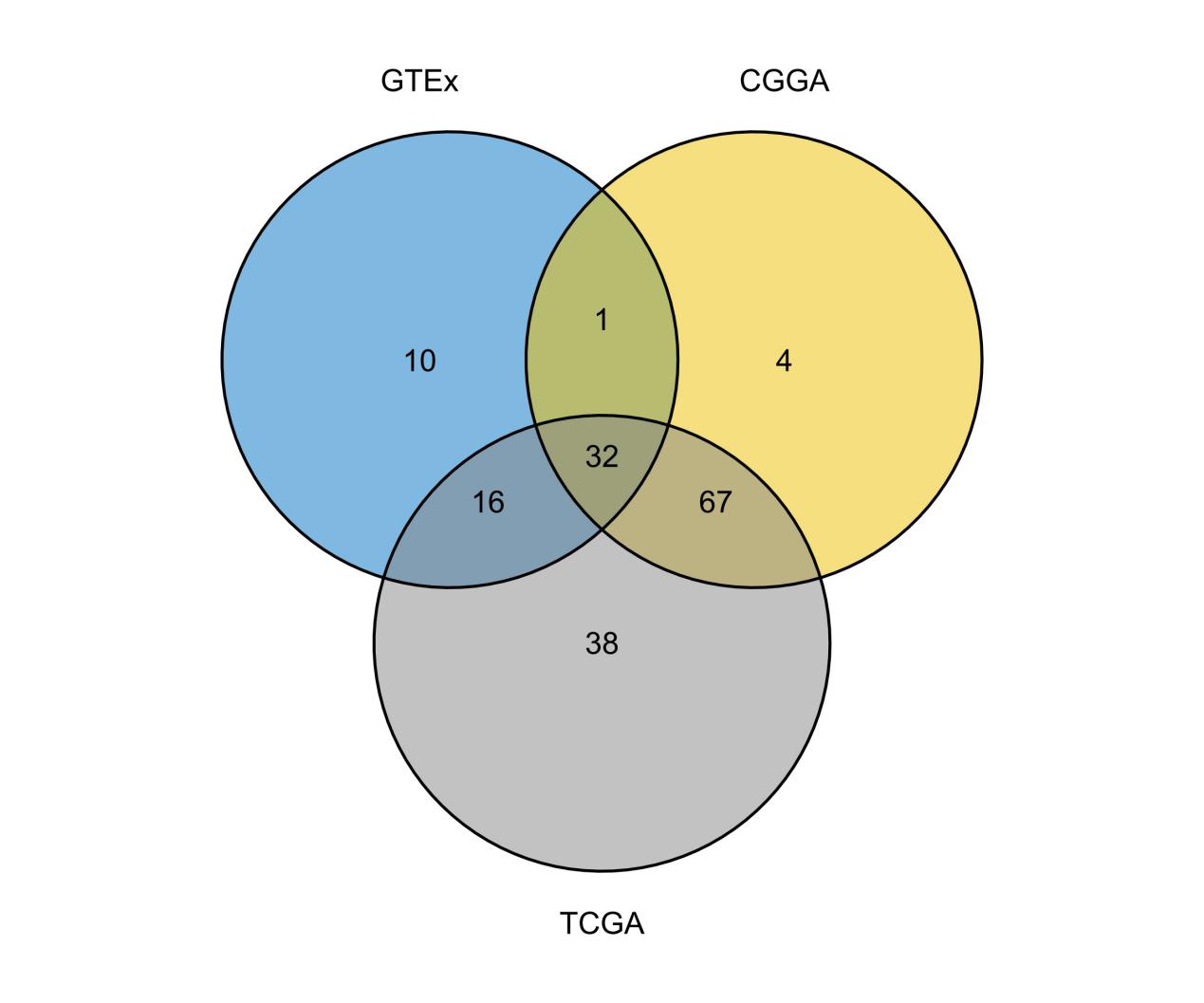


**S2. Kaplan–Meier survival analysis of glycosylation related gene signature in the TCGA cohort.**

**
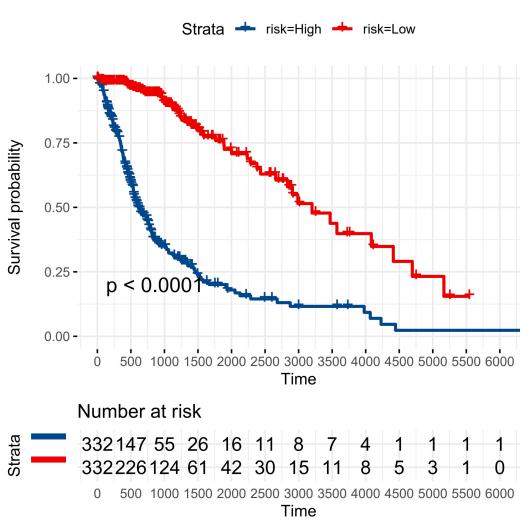
**

**S3. Kaplan–Meier survival analysis of the each and overall glycosylation related gene signature in the CGGA cohort.**

**A.CHPF2**

**
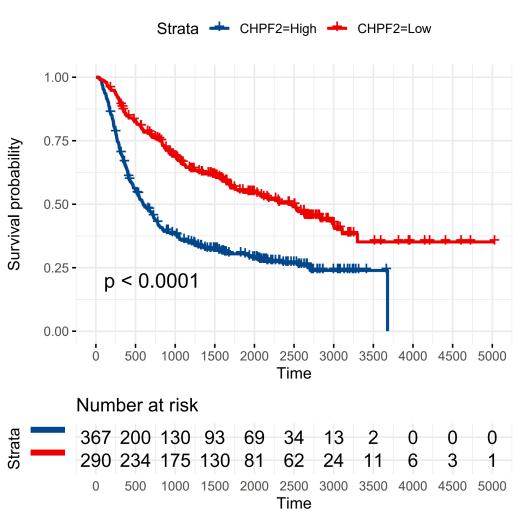
**

**B.COLGALT2**

**
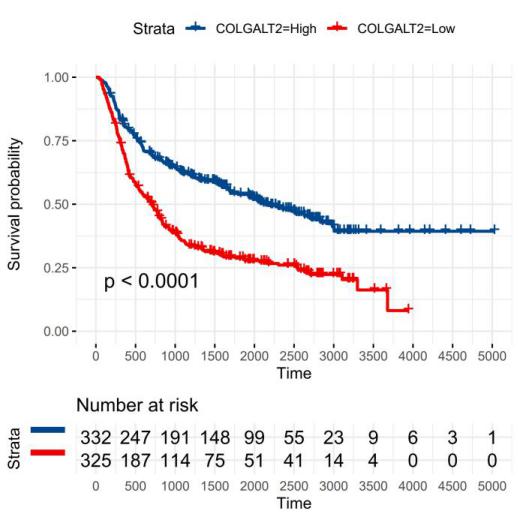
**

**C.EXT2**

**
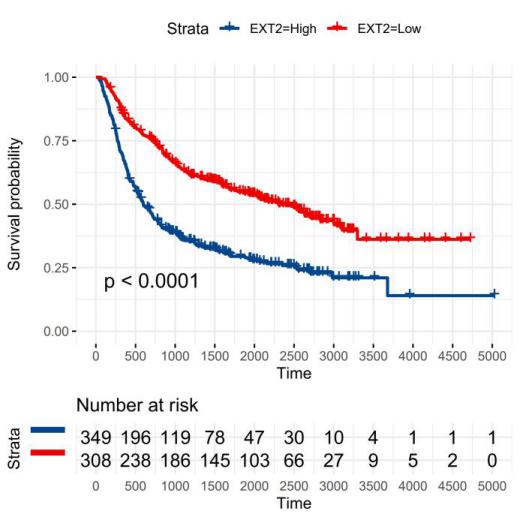
**

**D.GALNT13**

**
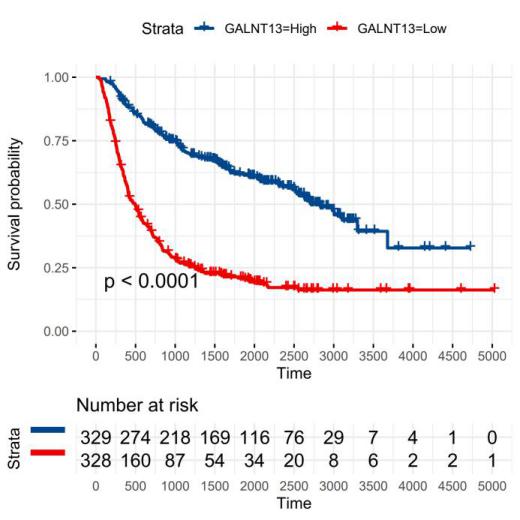
**

**E.PYGL**

**
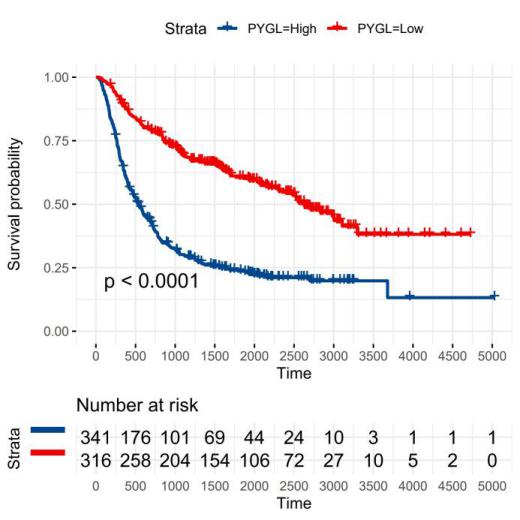
**

1. **Overall**

**
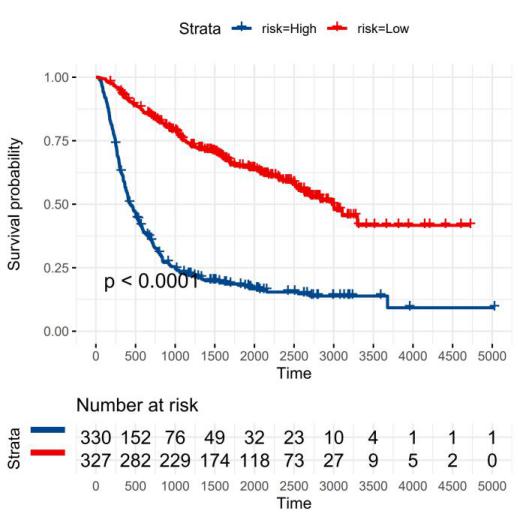
**

**S4. Kaplan–Meier survival analysis of the each and overall glycosylation related gene signature in the REMx cohort.**

**A.CHPF2**

**
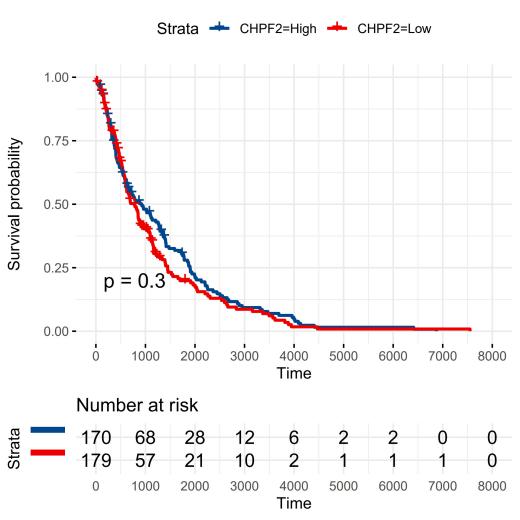
**

**B.COLGALT2**

**
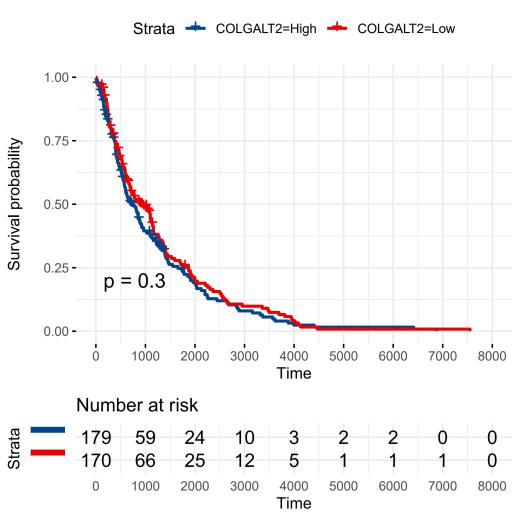
**

**C.EXT2**

**
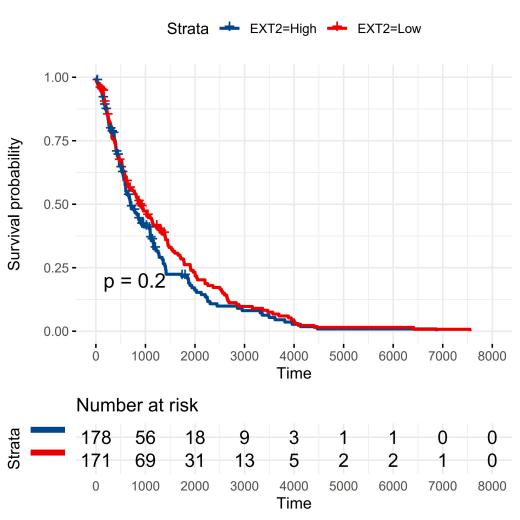
**

**D.GALNT13**

**
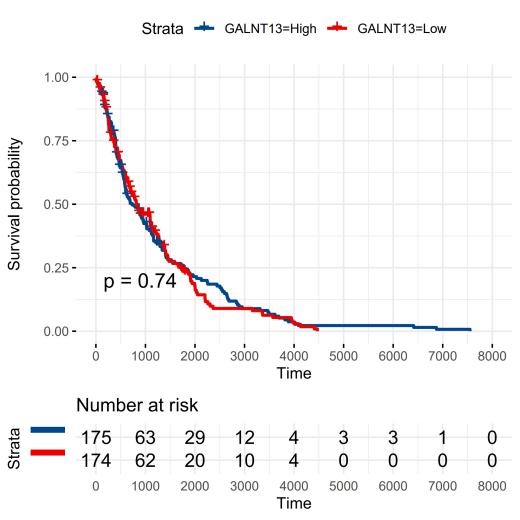
**

**E.PYGL**

**
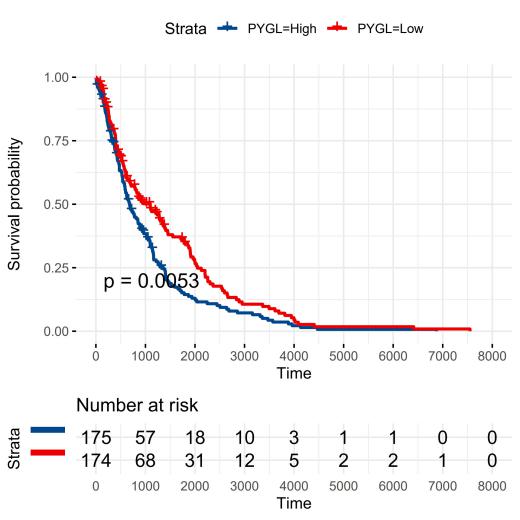
**

**F.Overall**

**
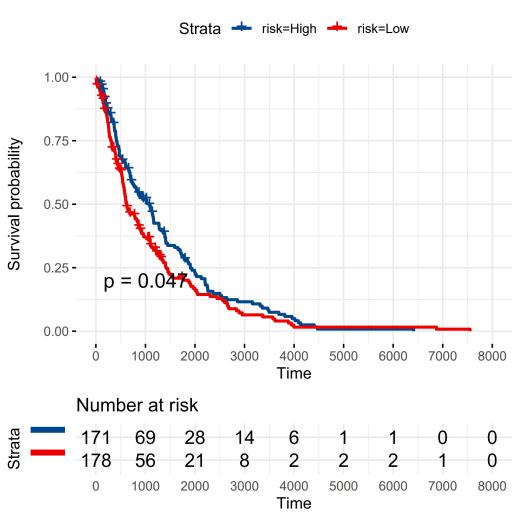
**

**S5. The heatmap was used to screen differential gene expression of 5 selected genes between the high- and low- risk groups at CGGA dataset.**

**
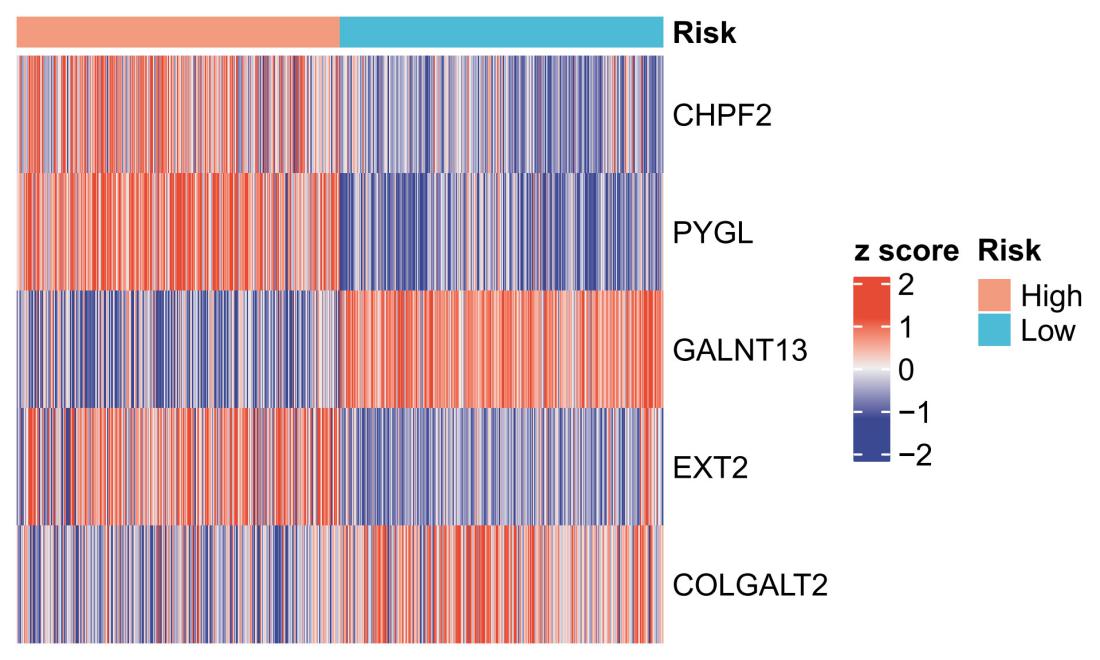
**

**S6. The heatmap was used to screen differential gene expression of 5 selected genes between the high- and low-risk groups at TCGA dataset.**


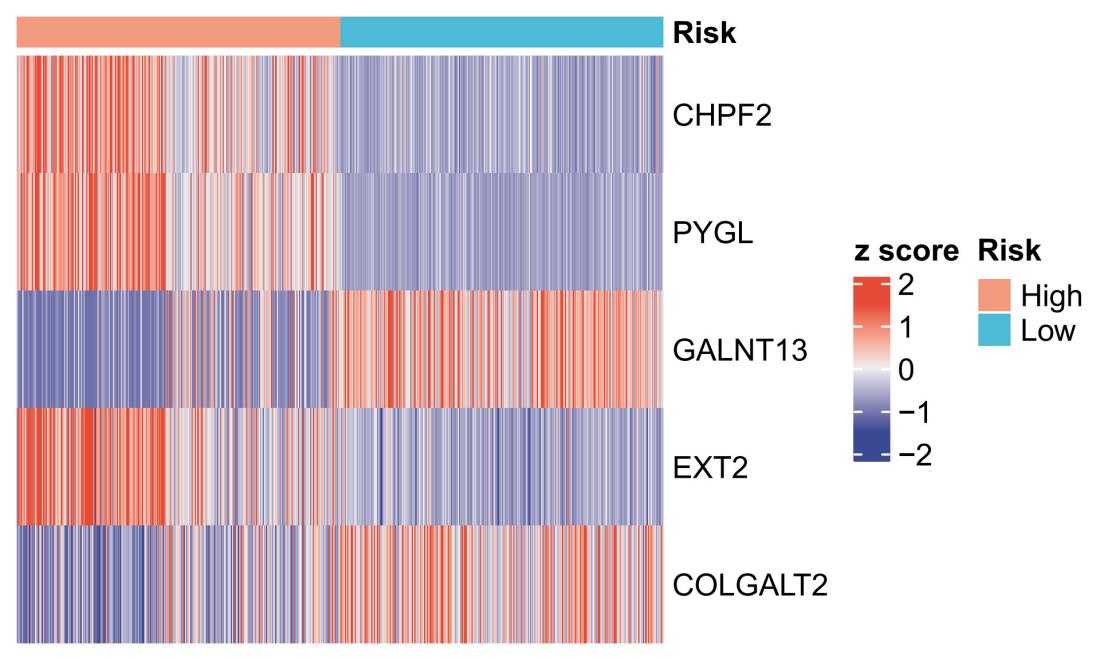


**S7. KEGG functional enrichment analysis of CGGA datasets.**


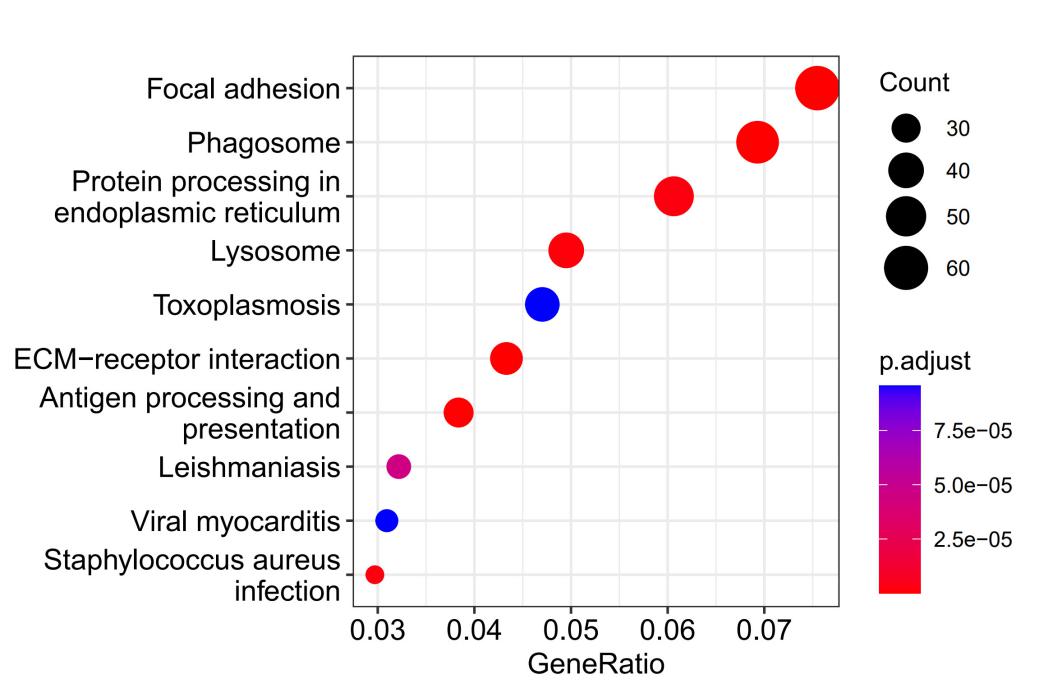


**S8. KEGG functional enrichment analysis of TCGA datasets.
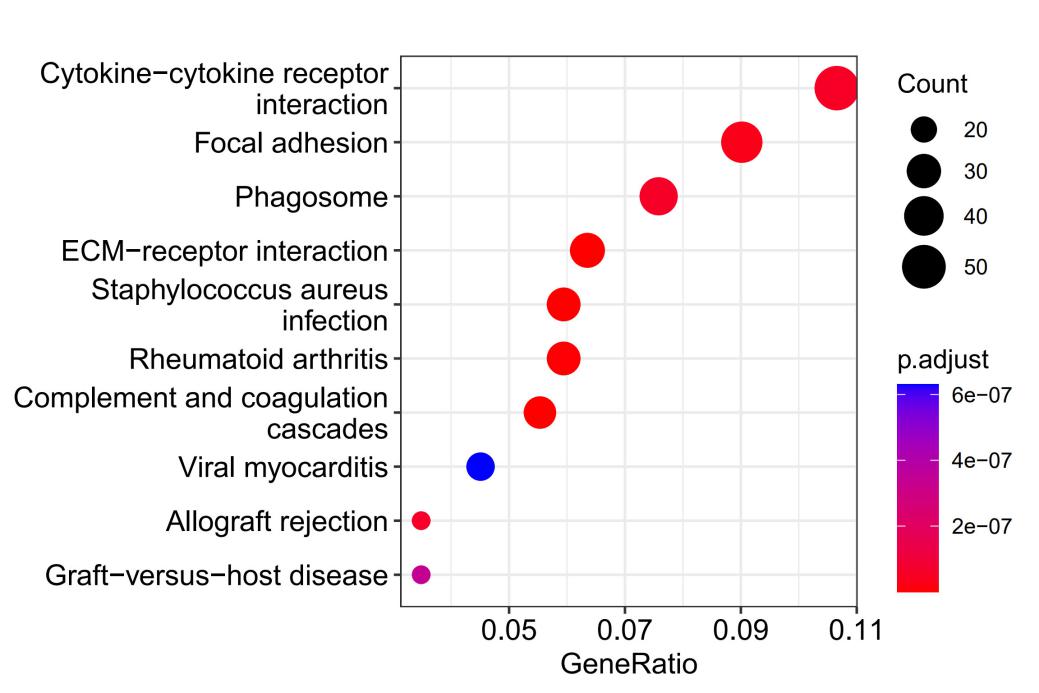
**
